# Exploiting physico-chemical properties in string kernels

**DOI:** 10.1186/1471-2105-11-S8-S7

**Published:** 2010-10-26

**Authors:** Nora C Toussaint, Christian Widmer, Oliver Kohlbacher, Gunnar Rätsch

**Affiliations:** 1 Center for Bioinformatics, Eberhard-Karls-Universität, Sand 14, 72076 Tübingen, Germany; 2 Friedrich Miescher Laboratory of the Max Planck Society, Spemannstr. 39, 72076 Tübingen, Germany

## Abstract

**Background:**

String kernels are commonly used for the classification of biological sequences, nucleotide as well as amino acid sequences. Although string kernels are already very powerful, when it comes to amino acids they have a major short coming. They ignore an important piece of information when comparing amino acids: the physico-chemical properties such as size, hydrophobicity, or charge. This information is very valuable, especially when training data is less abundant. There have been only very few approaches so far that aim at combining these two ideas.

**Results:**

We propose new string kernels that combine the benefits of physico-chemical descriptors for amino acids with the ones of string kernels. The benefits of the proposed kernels are assessed on two problems: MHC-peptide binding classification using position specific kernels and protein classification based on the substring spectrum of the sequences. Our experiments demonstrate that the incorporation of amino acid properties in string kernels yields improved performances compared to standard string kernels and to previously proposed non-substring kernels.

**Conclusions:**

In summary, the proposed modifications, in particular the combination with the RBF substring kernel, consistently yield improvements without affecting the computational complexity. The proposed kernels therefore appear to be the kernels of choice for any protein sequence-based inference.

**Availability:**

Data sets, code and additional information are available from http://www.fml.tuebingen.mpg.de/raetsch/suppl/aask. Implementations of the developed kernels are available as part of the Shogun toolbox.

## Background

String kernels are a powerful and popular tool for machine learning in computational biology. They have been successfully applied to numerous applications ranging from protein remote homology detection [1-3], to gene identification [4-6], to sub-cellular location prediction [7,8] to drug design [9,10]. The different kernel formulations commonly exploit the sequential structure of the sequences and by doing so can effectively eliminate implausible features, leading to improved results. When using string kernels on protein sequences, one key disadvantage is that prior knowledge about the properties of individual amino acids (AAs), e.g., their size, hydrophobicity, secondary structure preference, cannot be easily incorporated. While these properties can be learned implicitly by the machine learning methods if the training data sets are large enough, it would be advantageous to include this information in the sequence representation. The goal of this work is to combine the benefits of string kernels with the ones of physico-chemical descriptors for AAs. The main idea is to replace the comparison of substrings, which is computed during kernel computation, with a term that takes the AA properties into account. While this seems quite simple at first sight, it is less so, when considering *k*-mers instead of single AAs. The key insight is how to compute the kernels such that the beneficial properties of sequence kernels do not get lost. In particular, we would like that either the use of uninformative descriptors (e.g., each AA corresponds to a unit vector) or the choice of distinct kernel parameters reduces the new kernel to the original string kernel.

### String kernels for sequence classification

Kernels that have been proposed for classifying nucleic and amino acids can be divided into two main classes: (a) kernels describing the sequence content of sequences of varying length and (b) kernels for identifying localized signals within sequences of fixed length. The first class is typically used for classifying whole protein or mRNA sequences, while the second class is typically used to recognize a specific site in a window of fixed length sliding over a sequence.

#### *Kernels describing l-mer content*

The so-called* spectrum kernel* was first proposed for classifying protein sequences [11]:

  (1)

where** x, x′** are two sequences over an alphabet Σ, e.g. protein or DNA sequences.  is a mapping of the sequence** x** into a |Σ|*^l^*-dimensional feature-space. Each dimension corresponds to one of the |Σ|*^l^* possible strings *s* of length *l* and is the count of the number of occurrences of *s* in** x.** It is

 (2)

where **x**_[_*_i_*_:_*_i_*_+_*_l_*_]_ is the substring of length *l* of** x** at position *i*.

Several algorithms based on string or sparse data structures have been proposed to efficiently compute the above kernel and additional variants (for instance, with gaps, mismatches, mixed-order, etc.). The kernel in (1) can alternatively be written as

 (3)

 (4)

Here, we consider all pairs of substrings at any position in each of the two input sequences. This formulation has the benefit that it makes the comparison between the substrings more explicit, which is needed in the derivation of the extensions.

#### *Kernels for localized signals*

The spectrum kernel is less well-suited for identifying localized signals in sequences, since the information about the position of the substring in the input sequences is ignored, i.e. lost, during kernel computation. Several kernels have been proposed to address this issue. Most notably the* weighted degree (WD) kernel *[12] and the* oligo kernel *[13]. Both kernels work on sequences of fixed length *L* and count co-occurring substrings in both sequences at the same or similar position. We will use the WD kernel as representative for localized signal kernels. It is defined as

  (5)

where  is the weighting of the substring lengths. The WD kernel is quite related to the spectrum kernel formulation in (4), where we consider only the *l*-mers occurring at the same position, i.e., where *i* = *j*. The oligo kernel is similar in spirit but it also compares substrings at different positions.

#### *Incorporation of knowledge on AA properties*

In this work we propose modifications to existing string kernels that supplement the kernels’ beneficial properties by incorporating prior knowledge on physico-chemical and other properties of AAs. Previous work on incorporating prior knowledge has been either focused on using physico-chemical properties for single AAs, i.e., ignoring the sequential nature of the sequences (e.g., [14,15]), or took advantage of Blast or PSI-Blast profiles for improving spectrum kernels [2,3,16]. We propose a complementary approach of employing physico-chemical or other information to refine the similarity between two substrings used in most existing string kernels. We illustrate the usefulness of these modifications for both classes of string kernels on two problems: (a) the prediction of MHC-binding peptides as an example for localized signals and (b) protein fold classification as an example for *l*-mer content.

## Methods

### Idea

The string kernels described above (cf. (4),(5)) have in common that they compare substrings of length* l* between the two input sequences** x** and** x′**. The involved term  can equivalently be written as:

where  and .

 can be indexed by a substring *s* ∈ Σ*^l^* and is defined as , if  and 0 otherwise. For the sake of the derivation, let us consider , generating a simple encoding of the letters into |Σ|-dimensional unit vectors. It can be easily seen that we can rewrite the substring comparison as

The main problem of using Φ_1_ as the basis of substring comparisons, is that it ignores the relations between the letters in the alphabet. While this is a negligible problem for nucleotide sequences where each nucleotide is unique, it is important to consider relatedness between AAs. The main idea of this work is to replace Φ_1_ with a feature map Ψ that takes relations between the AAs into account. One way is to use physico-chemical descriptors of AAs, such as [17]. Alternatively, one may use AA substitution matrices for defining amino acid similarities, as e.g. done in [18]. The feature space is then not spanned by |Σ|*^l^* different combinations of letters, but by* D^l^,* where *D* is the number of properties used to describe the AA. This leads to the following kernel on AA substrings:

 (6)

Using the feature representation corresponding to this kernel, we can now recognize sequences of AAs that have certain properties (e.g. first AA: hydrophobic, second AA: large, third AA: positively charged, etc.):  There is a feature induced in the kernel corresponding to all combinations of products of features involving exactly one AA property per substring position. For instance, when considering products of the form (**x**_1,1_ + **x**_1,2_ +…+ **x**_1,_*_n_*) · (**x**_2,1_ +**x**_2,2_ +…+** x**_2,_*_n_*) · (**x**_3,1_ +** x**_3,2_ +…+** x**_3,_*_n_*), then we get* n*^3^ different monomials which each use exactly one of the *n* features from the three different groups. There are no monomials** x***_i_*_,_*_j_***x***_i_*_,_*_k_* for any *i* = 1,…,3 and *j*, *k* = 1,…,*n*.

If one wants to additionally allow the combination of several properties from every position, then the following two formulations are suitable: The first is based on the polynomial kernel:

  (7)

and the second on the RBF kernel:

  (8)

Both kernels induce a considerably richer feature space, which can be beneficial for accurate classification of sequences.

### AA substring kernel for localized string kernels

Replacing the substring comparison  with the more general formulation in (6), (7), or (8) together with an informed choice of features Ψ(*a*) for each letter* a* ∈ Σ (i.e. for each AA), directly implies a generalized form of the string kernels described above. For the WD kernel we can write:

  (9)

 is a linear combination of kernels and therefore a valid kernel [19]. Independent of the choice of AA substring kernel, the modified WD kernel can be computed efficiently, with a complexity comparable to that of the original.

Of particular interest is the* WD-RBF kernel,* i.e. the combination of the WD kernel and the RBF AA substring kernel:

 (10)

For* σ →* 0 and an encoding Ψ with Ψ(*a*) = Ψ(*b*) if and only if* a* = *b*, the WD-RBF kernel corresponds to the WD kernel: the RBF AA substring kernel will be one only if the substrings are identical, otherwise it will be zero.

#### *Relation to non-substring-based kernels*

When considering kernels for sequences of fixed length *L*, one may alternatively consider a representation of the sequence as vector of the physico-chemical properties of all sequence elements/AAs, i.e. . Then one may use a standard kernel to compute similarities between the sequences, as, e.g., done in [20]. When using the polynomial kernel as basis, this would lead to the following kernel:

 (11)

For the RBF kernel we get analogously,

  (12)

Please note that here we use the full sequence and do not separately consider subsequences. Both kernels consider higher order correlations between properties of the sequence at arbitrary position in the sequence. Hence, the sequential nature of the sequences is not fully taken into account (particularly important for long sequences).

### AA substring kernel for *l*-mer content string kernels

The AA substring kernels (6), (7), (8) can be combined with the spectrum kernel (1), (4) analogously to the combination with the WD kernel. For instance in combination with the RBF substring kernel, we arrive at:

  (13)

As before, for* σ →* 0, the above formulation is identical to the original spectrum kernel. A drawback of this approach is, however, that one now has to compute the substring comparisons for every pair of occurring substrings. Hence, the computational complexity, *O*(|**x**| · |**x′**|), is much higher than for the original spectrum kernel and makes this kernel impractical.

In order to reduce complexity we turn to modifications of the spectrum kernel: the* mismatch kernel *[21] and the* profile kernel *[2].

#### *The mismatch kernel*

While the spectrum kernel only considers pairs of identical *l*-mers, the mismatch kernel allows for some degree of mismatching. Instead of counting occurences of *l*-mers *s* in** x** it counts the occurences of *l*-mers that differ from *s* by at most *m* mismatches. The mismatch kernel is defined as

  (14)

and

  (15)

with ∅*_s_*(**x**_[_*_i_*_:_*_i_*_+_*_l_*_]_) = 1 if** x**_[_*_i_*_:_*_i_*_+_*_l_*_]_ belongs to the mismatch neighbourhood *N*_(_*_l_*_,_*_m_*_)_(*s*), i.e. differs from *s* in at most *m* positions. Otherwise, ∅*_s_*(**x**_[_*_i_*_:_*_i_*_+_*_l_*_]_) = 0. Thus, we can write alternatively:

 (16)

Combination of an AA substring kernel with the (*l*,*m*)-mismatch kernel limits comparisons to those *l*-mer pairs with at most 2*m* mismatches as opposed to all *l*-mer pairs for the spectrum kernel. Employing the mismatch tree data structure from [21], the generalized mismatch kernel can be calculated efficiently with a complexity of *O*(|Σ|*^m^l^m^*   (|**x**| + |**x′**|)). The (*l*, *m*)-mismatch tree is a tree representation of the feature space: each leaf represents a fixed *l*-mer feature *s*. In order to benefit from this feature space-based data structure, it suggests itself to apply the generalization to the feature map Φ_(_*_l_*_,_*_m_*_)_(**x**)***_s_*** (16). Plugging one of the AA substring kernels into (16) yields

  (17)

Rather than simply counting similar substrings this feature representation accounts for the degree of similarity: similar substrings contribute stronger than dissimilar ones. This strategy is particularly beneficial, when allowing many mismatches.

Once again, the combination with the RBF AA substring kernel, namely the* mismatch-RBF kernel,* is of particular interest. The corresponding feature map is defined as

  (18)

For* σ* → ∞ it corresponds to the mismatch feature map (16) since the RBF AA substring kernel will be one for all substring pairs.

#### *The profile kernel*

Just like the spectrum and the mismatch kernel, the profile kernel [2] was proposed for protein classification and remote homology detection. The main difference between the mismatch and profile kernel is the definition of the neighbourhood. For the profile kernel one uses the* positional mutation neighbourhood* of** x** based on blast or PSI-blast profiles *p*(**x_i_**, *k*) for each position *i* of** x** and for each letter *k* ∈ Σ:

  (19)

where δ defines the “radius” of the mutation neighbourhood [2]. Then the feature map and kernel, respectively, are defined as

  (20)

and

  (21)

In order to incorporate AA properties, we propose to modify (20) analogously to the mismatch kernel:

  (22)

The second term determines whether the substring is within the mutation neighbourhood and should be counted and the first term determines the contribution of the substring based on AA similarities. This kernel can be computed as efficient as the original profile kernel. Since the elements in the neighbourhood are weighted based on AA property similarity, the kernel may be able to take advantage of larger neighbourhoods.

The profile kernel is similar to the profile-based direct kernels described in [16] and similar ideas to incorporate AA properties can be applied there as well. The profile and mismatch kernel have, however, the advantage that they allow for an efficient computation using the data structures proposed in [2,22]. These data structures unfortunately are not applicable to the profile kernel formulations in [16].

### Experimental methods

#### *Data*

We evaluate the performance of the proposed kernels on two problems: the kernels for localized signals on MHC-peptide binding classification, and the kernels describing *l*-mer content on protein classification. For MHC-peptide binding experiments we utilized the IEDB benchmark data set from Peters* et al. *[23]. It contains quantitative binding data (IC_50_ values) of nonameric peptides with respect to various MHC alleles. Peptides with IC_50_ values greater than 500 were considered non-binders, all others binders. Protein classification data was taken from the supplementary material of [3]. This commonly used data set comprises 7,329 protein domains from 54 families. Corresponding profile information was taken from [http://cbio.mskcc.org/leslielab/software/string-kernels].

#### *Physico-chemical descriptors*

A wide range of physico-chemical descriptors of AAs have been published. Many of them can be obtained from the amino acid index database (AAIndex) [24]. Within this work we use three sets of descriptors: (1) five descriptors derived from a principal component analysis of 237 physico-chemical properties taken from the AAIndex [17] (*pca*), (2) three descriptors representing hydrophobicity, size, and electronic properties (*zscale*), and (3) 20 descriptors corresponding to the respective entries of the Blosum50 substitution matrix [25] (*blosum50*).

#### *Evaluation of string kernels for localized signals*

**Performance analysis.** Preliminary experiments on three human MHC alleles (A*2301, B*5801, A*0201) were carried out to analyze the performance of the different kernels WD (5), RBF (12), poly (11), WD-RBF (10), WD-poly (as WD-RBF, but with polynomial substring kernel) combined with different encodings (*pca, zscale, blosum50*). The alleles were chosen to comprise a small data set (A*2301, 104 examples) as well as a medium (B*5801, 988 examples) and a large (A*0201, 3,089 examples) data set from the IEDB benchmark [23]. Performances of the WD kernel and the WD-RBF kernel with* blosum50* encoding were subsequently analyzed on all 35 human MHC alleles contained in the IEDB benchmark. We used two times nested 5-fold cross-validation, i.e. two nested cross-validation loops, to (1) perform model-selection over the kernel and regularization parameters (inner loop), (2) estimate the prediction performance (outer loop) (see, e.g., page S30 of the supporting online material of [26]). Performance is measured by averaging the area under the ROC curve (auROC).

**Learning curve analysis.** The performance dependence on the amount of training data was analyzed on allele A*0201 in 100 runs of two times nested 5-fold cross-validation to average over different data splits to reduce random fluctuations of the performance values. Performance is measured by averaging the area under the ROC curve (auROC). In each run, thirty percent of the available data was used for testing. From the remaining data training sets of different sizes (20, 31, 50, 80, 128, 204, 324, 516, 822, 1,308) were selected randomly.

#### *Evaluation of string kernels describing l-mer content*

**Mismatch kernel.** For the comparison of the mismatch kernel and the mismatch-RBF kernel, protein classification data and experimental setup were taken from the supplementary material of [3]. The ROC_50_ score, i.e. the area under the ROC curve computed up to the first 50 false positives, is used as performance measure.

**Profile kernel.** For the comparison of the profile kernel and the profile-RBF kernel, protein classification data and experimental setup were taken from the supplementary material of [3]. Corresponding PSI-blast profiles were taken from [27]. The ROC_50_ score is used as performance measure.

#### *SVM computations*

All SVM computations were performed using the Matlab interface of the freely available large scale machine learning toolbox Shogun [28]. All used kernels are implemented as part of the toolbox.

## Results and discussion

The main goal of this work is the methodological improvement of existing string kernels by incorporation of prior knowledge on AA properties. In order to analyze the benefits of the proposed modifications we conducted performance comparisons between the original and the modified string kernels.

### String kernels for localized signals

**Figure 1:**
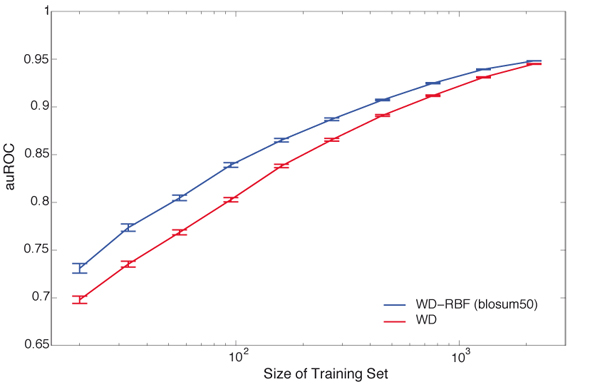
Learning Curve Analysis on MHC allele A*0201. Shown are areas under the ROC curves averaged over 100 different test splits (30%) and for increasing numbers of training examples (up to 70%). The training part was used for training and model selection using 5-fold cross-validation.

The prediction of MHC-binding peptides is one of the major problems in computational immunology, highly relevant for rational vaccine design. MHC-I molecules bind small peptides derived from intracellular proteins and present them on the cell surface for surveillance by the immune system. Given a set of peptide sequences one would like to predict whether they bind to a certain MHC-I molecule. Since the majority of binders are of length nine, the application of kernels for localized signals suggests itself. For a preliminary analysis we chose three human MHC alleles from the IEDB benchmark data set: A*2301 (104 peptides), B*5801 (988 peptides), and A*0201 (3,089 peptides). The performance of various kernels utilizing sequential structure only (WD kernel), AA properties only (RBF, poly), and a combination of both (WD-RBF, WD-poly) was validated on these alleles. We used three different encodings of AA properties. Cross-validation results are given in Table 1.Best performance is achieved by a different kernel type for each of the alleles: poly (*pca*) for A*2301, RBF (*blosum50*) for B*5801 and WD-RBF (*blosum50*) for A*0201. The latter performs second-best on A*2301 and B*5801. As for the benefits of the modification of the WD kernel, the WD-poly and WD-RBF kernels outperform the WD kernel in 17 out of 18 cases. From Table 1 we can observe the trend that the kernels that use AA properties benefit more for smaller datasets. To validate this hypothesis, we performed a learning curve analysis for WD and WD-RBF (*blosum50*) on A*0201, the allele with the highest number of peptides in the IEDB benchmark data set. Figure 1 shows the mean auROCs with confidence intervals  over 100 cross-validation runs. We can clearly observe that the fewer examples are available for learning, the stronger is the improvement of the WD-RBF kernel over the WD kernel. Intuitively this makes sense, as the more data is available, the easier it will be to infer the relation of the AAs from the sequences in the training data alone.

**Table 1:** Performances of kernels utilizing sequential structure and/or AA properties on three MHC alleles

KERNEL	A*2301		B*5801		A*0201	
	**auROC **	**(std) **	**auROC **	**(std) **	**auROC **	**(std)**

WD	0.7307	(0.0900)	0.9314	(0.0279)	0.9485	(0.0076)

Poly (*pca*)	** 0.8363 **	(0.0808)	0.9428	(0.0336)	0.9354	(0.0111)
Poly (*zscale*)	0.7964	(0.0727)	0.8778	(0.0637)	0.9052	(0.0070)
Poly (*blosum50*)	0.8220	(0.0442)	0.4948	(0.0560)	0.4729	(0.0246)

RBF (*pca*)	0.8277	(0.0904)	0.9396	(0.0303)	0.9345	(0.0114)
RBF (*zscale*)	0.7847	(0.0787)	0.9235	(0.0347)	0.9157	(0.0072)
RBF (*blosum50*)	0.8204	(0.0864)	0.9509	(0.0317)	**0.9520 **	(0.0072)

WD-Poly (*pca*)	0.7879*	(0.0858)	0.9406*	(0.0319)	0.9495*	(0.0084)
WD-Poly (*zscale*)	0.7983*	(0.0902)	0.9499*	(0.0348)	0.9483	(0.0073)
WD-Poly (*blosum50*)	0.8307*	(0.1077)	0.9491*	(0.0224)	0.9490*	(0.0070)

WD-RBF (*pca*)	0.8133*	(0.0806)	0.9510*	(0.0265)	0.9486*	(0.0051)
WD-RBF (*zscale*)	0.7782*	(0.1222)	0.9487*	(0.0434)	0.9500*	(0.0074)
WD-RBF (*blosum50*)	0.8312*	(0.0993)	0.9571*	(0.0265)	0.9503*	(0.0067)

auROCs and standard deviation were determined in two times nested 5-fold cross-validation. Best (bold) and second-best (underlined) performances per MHC allele are highlighted. An asterisk marks performance improvement due to the proposed modifications.

**Figure 2:**
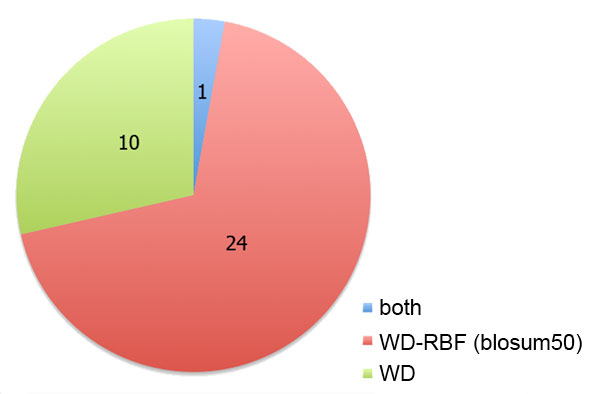
**Performance of WD and WD-RBF (*blosum50*) kernels on human MHC alleles from the IEDB benchmark data set.** The pie chart displays the number of alleles for which the WD (green) and the WD-RBF (red) performed best, respectively, and the number of alleles for which they performed equally (blue).

The preliminary analysis showed the WD-RBF kernel with* blosum50* encoding to perform best. For a more comprehensive comparison, performance of WD and WD-RBF (*blosum50*) kernels were assessed on all 35 human MHC alleles from the IEDB benchmark. For 24 alleles WD-RBF outperforms WD (Fig. 2). This is significant with respect to the binomial distribution (p-Value = 0.0083).

Finally, we compare our results with the ones obtained using a multi-task learning (MTL) method for MHC classification described in [9]. Here, the authors used two kernels, one to define the similarity between examples and one to define the similarity between tasks. They report an auROC of 90.3% using two string kernels. When using the WD-RBF for computing the similarity between the examples, we can slightly improve upon their performance to 90.5% (data splits and model selection as in [9]). Hence, the AA property-enhanced kernels once more have a slight, but consistent advantage over the base-line kernels. Besides the performance improvement, the modified WD kernel allows, at least theoretically, for the extraction of biological insights: employing an analysis method analogous to [29] individual patterns of AA properties that are relevant for the classification can be extracted.

### String kernels describing *l*-mer content

To show that also the modification of kernels for describing *l*-mer content of sequences has desirable properties, we chose the problem of protein remote homology detection. Here, the task is to classify proteins into folds, super-families or families based on their sequence. This problem has been previously tackled in a series of papers in [11,21,22] which suggested the spectrum kernel, followed by the mismatch kernel and finally the profile kernel. The profile kernel already uses AA similarities based on blast or PSI-blast profiles which lead to significant improvements. Here, we would like to illustrate that using the AA property-enhanced versions of these kernels can still lead to an improvement. We chose the family classification task for this analysis since it was considered in all mentioned previous studies.

Table 2 shows the average auROC_50_ score over the 54 families we obtained for the family classification problem. Furthermore, the number of times for which each method outperforms its counterpart is displayed. We compare the* spectrum kernel *[11] with the* spectrum-RBF kernel* as in (13) with* pca* features, the* mismatch kernel *[22] with the* mismatch-RBF kernel* as in (18); and the* profile kernel *[21] with the* profile-RBF kernel* as in (22). For all three cases we find that the AA property-enhanced kernels improve the original kernels. For* spectrum* and* mismatch* kernel these improvements are significant with respect to the binomial distribution. Most notably, the performance of the spectrum kernel can be drastically improved from 15.1% to 42.1%. However, the more sophisticated the original kernel already is, the smaller is the improvement that can be achieved by using additional AA property information.

**Table 2:** Comparison of kernels for *l*-mer content with their AA-property enhanced counterparts.

Method	auROC_50_	#Wins
Spectrum (*l* = 5)	15.2%	7/54
Spectrum-RBF (*l* = 5, *σ* = 1)	42.1%	45/54

Mismatch (*l* = 5, *m* = 1)	42.3%	13/54
Mismatch-RBF (*l* = 5,*m* = 1, *σ* = 1)	43.6%	36/54

Profile (*l* = 5, *τ* = 7.5)	82.1%	3/54
Profile-RBF (*l* = 5,*τ* = 7.5, *σ* = 100)	82.2%	10/54

Comparison of the three kernels proposed in [11,21,22], with their AA-property enhanced counterparts for remote homology detection of 54 protein families. auROC_50_ is the average auROC_50_ score and #Wins the number of families for which each method outperforms its counterpart (Spectrum vs. Spectrum-RBF, Mismatch vs. Mismatch-RBF, Profile vs. Profile-RBF). The kernels taking advantage of AA properties lead to a higher average accuracy in all three cases (p-Values: 6.92   10^−8^ for* spectrum,* 0.0045 for *mismatch,* and 1.0 for* profile* kernels). For *l* and *τ* we use the published parameter settings. For *σ* we chose the best result among *σ* = {0.1,1,10,100,1000}.

In summary, in our experiments we can observe that the newly proposed kernels lead to consistently better performances than the string kernels on AA sequences as well as the non-substring kernels.

## Conclusions

We have proposed new kernels that combine the benefits of physico-chemical descriptors for amino acids with the ones of string kernels. String kernels are powerful and expressive, yet one needs sufficiently many examples during training to learn relationships between amino acids in the very high dimensional space induced by the string kernel. Standard kernels based on physico-chemical descriptors of amino acids, on the other hand, cannot exploit the sequential structure of the input sequences and implicitly generate many more features, numerous of which will be biologically implausible. Here, one also needs more examples to learn which subset of features is needed for accurate discrimination, especially for longer protein sequences.

We could show that the proposed modifications of the WD kernel yield significant improvements in the prediction of MHC-binding peptides. As expected, the improvement is particularly strong when data is less abundant. For protein remote homology detection AA property-enhanced kernels can also lead to significant performance improvements. For the most sophisticated kernels using blast or PSI-blast profiles, however, information about the similarities of AAs can already be derived from the profiles and the improvement is marginal.

Overall, our experiments demonstrate that the proposed kernels indeed lead to a better performance than string kernels and non-substring kernels. These improvements are not major, but consistent. It has to be noted that a big difference between the previously proposed kernels and the proposed kernels cannot be expected: The proposed kernels essentially work on subsets of the features of previously proposed kernels and the improvements that we observe mainly come from the SVM’s degraded performance when including uninformative features (which typically is not very pronounced).

In summary, the proposed modifications, in particular the combination with the RBF AA substring kernel, consistently yield improvements without seriously affecting the computing time (except for the Spectrum-RBF kernel). In all formulations, the original string kernel formulation can be recovered by appropriately choosing *σ*. Hence, when *σ* is included in model selection, the performance of the proposed kernels should be at least as good as the original string kernels. We therefore believe that the proposed kernels should be preferred over the original formulations for any protein sequence classification task.

## List of abbreviations used

AA: amino acid; MHC: major histocompatibility complex; SVM: support vector machine; WD: weighted degree.

## Competing interests

The authors declare that they have no competing interests.

## Authors' contributions

NCT and GR conceived and designed the project. NCT prepared the data, implemented the kernels, performed experiments and drafted the manuscript. CW performed the MTL experiments and contributed to the preparation of manuscript. OK contributed to the discussion and helped writing the manuscript. GR supervised the project, implemented and performed experiments and contributed to the manuscript.
